# Facts Tell, Stories Sell? Assessing the Availability Heuristic and Resistance as Cognitive Mechanisms Underlying the Persuasive Effects of Vaccination Narratives

**DOI:** 10.3389/fpsyg.2022.837346

**Published:** 2022-03-07

**Authors:** Lisa Vandeberg, Corine S. Meppelink, José Sanders, Marieke L. Fransen

**Affiliations:** ^1^Behavioural Science Institute, Radboud University, Nijmegen, Netherlands; ^2^Centre for Language Studies, Radboud University, Nijmegen, Netherlands; ^3^Amsterdam School of Communication Research, University of Amsterdam, Amsterdam, Netherlands

**Keywords:** childhood immunization, narrative persuasion, availability heuristic, cognitive resistance, vaccination attitudes, attitude certainty, vaccine hesitancy, belief consistency

## Abstract

Online vaccine-critical sentiments are often expressed in appealing personal narratives, whereas vaccine-supporting information is often presented in a non-narrative, expository mode describing scientific facts. In two experiments, we empirically test whether and how these different formats impact the way in which readers process and retrieve information about childhood vaccination, and how this may impact their perceptions regarding vaccination. We assess two psychological mechanisms that are hypothesized to underlie the persuasive nature of vaccination narratives: the availability heuristic (experiment 1, N = 418) and cognitive resistance (experiment 2, *N* = 403). The results of experiment 1 showed no empirical evidence for the availability heuristic, but exploratory analyses did indicate that an anti-vaccination narrative (vs. expository) might reduce cognitive resistance, decrease vaccination attitudes and reduce attitude certainty in a generally pro-vaccination sample, especially for those who were more vaccine hesitant. Preregistered experiment 2 formally tested this and showed that not narrative format, but prior vaccine hesitancy predicts cognitive resistance and post-reading attitudes. Hesitant participants showed less resistance toward an anti-vaccine text than vaccine-supporting participants, as well as less positive post-reading attitudes and attitude certainty. These findings demonstrate belief consistency effects rather than narrative persuasion, which has implications for scientific research as well as public health policy.

## Introduction

Childhood immunization has drastically declined the occurrence of vaccine-preventable diseases such as measles. Nevertheless, parents in Western societies are increasingly hesitant about vaccinating their children (Omer et al., 2009; He et al., 2022). “Vaccine hesitant” generally refers to people who do not fall into the polarized categories of unquestioning vaccine acceptors or refusers, but are placed on the continuum between these poles including those who are ambivalent, experience doubts or concerns, delay vaccination, or accept only some vaccines (Leask et al., 2012; Smith, 2017; Stasiuk et al., 2021). Vaccine hesitancy poses an enormous threat to global health (World Health Organization, 2019).

When deciding on childhood vaccination, parents want balanced information about the benefits and harms, but they experience difficulty in finding unbiased information (Ames et al., 2017). Ongoing debates about vaccinations are confusing to parents, which may lead them to question and re-evaluate their choices (Downs et al., 2008). Although parents perceive health professionals as important sources of information (Ames et al., 2017), they are more likely to turn to the internet (Downs et al., 2008).

On the internet, parents are likely to encounter vaccine-critical information that is not based on scientific evidence (Davies et al., 2002). Such vaccine-critical information is frequently presented in an appealing storytelling format describing parents' negative experiences with vaccination (e.g., Kata, 2010; Sanders et al., 2019). Since personal narratives are known to be a persuasive format (Braddock and Dillard, 2016), hesitant parents' perceptions of vaccine safety are considered to be easily influenced toward negative attitudes regarding vaccination. Attempting to counter such societal developments, professional health communicators have started developing narrative approaches to encourage vaccine-positive attitudes.

To gain insight into the mechanisms underlying the impact of vaccination narratives, research has mainly focused on affective mechanisms (Wroe et al., 2005; Betsch et al., 2010; Sprengholz and Betsch, 2020). However, cognitive processes may play an important role in the formation and change of vaccination-related beliefs (Miton and Mercier, 2015). We address this by studying two cognitive mechanisms that might affect how people process, retrieve, and perceive information from online vaccination narratives; the *availability heuristic* and *cognitive resistance*. Experiment 1 compares a narrative vaccination message with a non-narrative, expository message to test whether it evokes the availability heuristic and to explore whether it elicits other mechanisms (e.g., less resistance against the message) and outcomes (e.g., shifts in attitude certainty). Experiment 2 was designed to further examine the exploratory findings from experiment 1, specifically aimed at examining a mediating role of cognitive resistance and a moderating role of prior vaccine hesitancy in the potentially persuasive effects of anti-vaccination narratives. This research provides an empirical test of two cognitive mechanisms that are hypothesized to underlie the impact of vaccination narratives on individuals' perceptions regarding vaccines, and examines whether pre-reading vaccine hesitancy is a boundary condition for such narrative effects.

## Theoretical Background

### Vaccine Information on the Internet

Parents seeking vaccine information on the internet are likely to encounter non-scientific information with a vaccine-critical sentiment (Davies et al., 2002; Jolley and Douglas, 2014; Guidry et al., 2015). As anti-vaccination sources appear to be effective communicators (Lutkenhaus et al., 2019b; Johnson et al., 2020), this can hinder the dissemination of evidence-based information supporting vaccines. Experimental evidence shows that brief exposure (0.5–10 min) to online information highlighting the potential harm of vaccines or supporting anti-vaccine conspiracy theories negatively impacts people's risk perceptions regarding vaccines and intentions to vaccinate (Betsch et al., 2010; Jolley and Douglas, 2014). Also, network analyses show that social media populations with anti- (vs. pro-) vaccine standpoints are more effective in reaching and communicating with the vaccine hesitant population (Johnson et al., 2020); also, pro-vaccine facts and figures hardly spill over to other communities, whereas vaccine myths do (Lutkenhaus et al., 2019b). Interestingly, evidence shows that the anti-vaccine discourse generally offers a wide variety of—potentially attractive—claims that are critical or negative about vaccines (e.g., Zimmerman et al., 2005; Johnson et al., 2020; Stasiuk et al., 2021), whereas the pro-vaccination discourse tends to be more monothematic in its approach (Johnson et al., 2020; Meppelink et al., 2021; Stasiuk et al., 2021).

The discourse on the opposite ends of the vaccine debate is not only different in terms of reach and thematic content, but also in terms of format or genre. Online texts containing anti-vaccine sentiments often use storytelling formats describing, for instance, parents' negative experiences with vaccination (Kata, 2010; Guidry et al., 2015; Sanders et al., 2019; Haase et al., 2020). Such anti-vaccination narratives are highly appealing because they often use archetypical roles to describe the parent's experiences in a “hero's journey” template and because they place a strong sense of agency on skeptical and refusing parents (Sanders et al., 2019). Texts representing pro-vaccine sentiments, on the other hand, regularly present information in an expository format, using impersonal mode, describing facts, figures, statistics, and displaying scientific evidence (Guidry et al., 2015; Lutkenhaus et al., 2019a,b; Sanders et al., 2019).

### Narrative Persuasion

It is likely insufficient to counter the persuasive rhetorical appeals in anti-vaccine messages by using mere factual refutational strategies (Davies et al., 2002). In line with this argument, healthcare providers report that the most effective way to convince vaccine-skeptical parents is to share their personal vaccine choices for their own children and their personal experiences with vaccine safety (Kempe et al., 2011). Consequently, storytelling is proposed as a potentially effective narrative intervention to improve evidence-based communication and stimulate immunization (Cawkwell and Oshinsky, 2016).

Narratives could help prevent the audience from reacting negatively to messages about a controversial topic. Stories about personal experiences are more readily digestible than argumentative, generic expositions and therefore pose fewer obstacles for a broad audience, including people with high and low reading and health literacy skills (Boeijinga et al., 2017a). In the context of health communication, a message is considered a narrative if it has an identifiable structure from start to finish, between which a situation unfolds, events take place, and a problem is addressed (Hinyard and Kreuter, 2007). It is also typical that a character—often an “I”-narrator—experiences the events and describes them from her or his own perspective (de Graaf et al., 2012). When readers (or listeners, viewers) are “transported” into the story, they are neither motivated nor able to properly perceive any guiding and moralizing intentions of the narrative (Green, 2006). Additionally, recognizable story characters with comprehensible goals and achievable solutions can be relevant role models for their target group (Hoeken et al., 2016; Boeijinga et al., 2017b) and arouse interest through specific story details that lead to deeper processing (Bernstein, 1955).

Experimental studies investigating the persuasive effects of pro- and anti-vaccination narratives so far show mixed evidence. On the one hand, evidence suggests that personal vaccination narratives are persuasive. For instance, research shows that samples of various individual vaccination narratives describing vaccine adverse events affect people's risk perceptions and vaccination intentions (Betsch et al., 2011; Haase et al., 2020). Also, personal narratives promoting adult vaccinations have more impact on people's risk perceptions and intentions to vaccinate than objective statistics promoting vaccination (Wit et al., 2008). On the other hand, evidence indicates that vaccination narratives are not necessarily more persuasive. For instance, studies on science-based vaccination narratives show that narratives aimed at correcting misinformation do not work (Sangalang et al., 2019; Kuru et al., 2021) or can even backfire (Nyhan et al., 2014). Yet other research suggests that combining narrative with statistical evidence in pro- vaccination messages has a greater impact on risk perceptions and intentions than messages presenting either narrative or statistical evidence (Nan et al., 2015).

Given these mixed findings, it is important to further examine whether and how narratives may shape vaccine decisions. Inspired by scholars arguing that vaccine decisions are sensitive to flaws and shortcuts in people's reasoning (e.g., Ball et al., 1998), we first test whether vaccination narratives might elicit the availability heuristic.

### The Availability Heuristic in Vaccine Decisions

Decisions regarding childhood vaccines are often insufficiently informed (Lehmann et al., 2017) and deliberations on the decision against vaccines demonstrably suffer from a variety of reasoning flaws (Jacobson et al., 2007). An explanation is that these decisions rely on the assessment of risk (Brewer et al., 2007)—both the risk of obtaining a vaccine preventable disease and the risk of obtaining vaccine side effects. Information about the risks of vaccines and vaccine preventable diseases is often difficult to understand, incomplete, or conflicting, resulting in uncertainty (Serpell and Green, 2006). When people make decisions under uncertainty, they are often susceptible to heuristics and biases. People rely on heuristics (“cognitive shortcuts”) when assessing probabilities by reducing complex mental operations to simplified judgmental tasks (Tversky and Kahneman, 1974). Such heuristics lead to numerous biases that affect people's decisions and regularly lead to erroneous judgment (Tversky and Kahneman, 1974).

Following this reasoning, vaccine decisions have been proposed to be prone to heuristics and biases (Ball et al., 1998; MacDonald et al., 2012; Niccolai and Pettigrew, 2016). Content analyses and surveys indeed support an association between various biases and vaccine decisions (e.g., Asch et al., 1994; Zimmerman et al., 2005; DiBonaventura and Chapman, 2008; Brown et al., 2010). One “usual suspect” that has been assumed to underlie vaccine decisions, especially when these are informed by narrative information, is the availability heuristic. The availability heuristic is defined as a mental strategy that is employed when people estimate the probability of an event based on how easily an instance of such an event comes to mind (Tversky and Kahneman, 1973). Thus, if people use this heuristic when assessing the risk of vaccine-preventable diseases like measles, their risk estimations will be higher when a measles-case is easily available from their memory vs. when it is more difficult to retrieve. Analogously, the perceived risk of vaccine-adverse events is affected by the mental availability of such an event, thus whether it is easy or difficult to access an instance of a child suffering from serious vaccine side effects.

The availability heuristic has been proposed to underlie vaccine decisions (Ball et al., 1998; Omer et al., 2017) and stimulate vaccine hesitancy (Jacobson et al., 2015), because it biases toward memories of vaccine adverse events (Stasiuk et al., 2021) and thereby results in increased vaccine risk perceptions (Serpell and Green, 2006; MacDonald et al., 2012). Several scholars have furthermore proposed that particularly vaccination information in a narrative format should induce an availability heuristic (Serpell and Green, 2006; Wit et al., 2008; Kuru et al., 2021). The rationale behind this is that the experiences described in a narrative format (vs. non-narrative vaccine information) present information in an appealing, vivid, and salient manner (Betsch et al., 2010, 2011). As narrative events are more salient and thereby likely more easily retrievable from memory compared to non-narrative information, their probability (e.g., in terms of risk perceptions associated with the described event) will be overestimated when an availability heuristic is adopted. Based on the presented arguments, we hypothesize that vaccination narratives lead to a more pronounced manifestation of the availability heuristic than non-narrative expositories that take a more generic, informative stance (Berman and Katzenberger, 2004):

*H1. Narrative texts about vaccination will lead to (a) greater experienced ease of retrieval and (b) increased risk perceptions compared to expository texts about vaccination*.

Vaccine-supporting information on the internet is often presented in a narrative format, whereas vaccine-critical information is often presented in a non-narrative, expository manner. As a result, message format and message content are usually confounded in real-life communication about vaccination. Several scholars have argued that an availability heuristic in vaccine decisions is likely driven by the *vaccine-critical content* of these events, rather than the format in which they are presented. For instance, vaccine adverse events might be highly available in our collective memory because their occurrence has increased (as more vaccines are being administered) relative to the decrease of vaccine-preventable diseases (Omer et al., 2017). Vaccine adverse events might also be more available because negative (vs. positive) portrayals of vaccination are more intuitive and thereby more likely to spread (Miton and Mercier, 2015).

To address any confound between message content (vaccine adverse events) and message format (narrative), we unravel the two by investigating what it is that may drive the availability heuristic. If the narrative format indeed drives an availability heuristic, risk perceptions should increase based on the described situation. That is, risk perceptions should reflect a higher estimated probability *of the described event*, regardless of whether the event describes vaccine adverse effects (increasing risk perceptions of the vaccine) or symptoms of a vaccine preventable disease (increasing risk perceptions of the disease). We therefore present texts focusing on the negative effects of vaccines (referred to as anti-vaccine content) as well as texts focused on the negative effects of a vaccine-preventable disease (referred to as pro-vaccine content) in the various formats. We specifically examine whether a potential availability heuristic not only manifests for anti- but also for pro-vaccine narratives.

*RQ1: Does text content (anti- vs. pro-vaccine) affect the relationship between narratives and (a) ease of retrieval and (b) risk perception?*

## Experiment 1

The purpose of experiment 1 was two-fold. First, we aimed to empirically test whether the availability heuristic explains the effectiveness of narrative vaccination messages vs. non-narrative expositories with similar arguments. Second, we aimed to examine a potential role of anti- vs. pro-vaccine content in this relation. Additionally, since previous research suggests that narratives can result in less critical message processing and more story-consistent beliefs (e.g., Green and Brock, 2000), we included various exploratory variables, including resistance toward the message, attitudes toward vaccination, and attitude certainty.

## Materials and Methods

### Design and Participants

The experiment consisted of a 2 (format: narrative/expository) ^*^ 2 (content: pro/anti) between-subjects design. Participants were recruited through the scientific crowdsourcing community Prolific Academic to take part in an online experiment (for more information about the general Prolific Academic population, see Prolific, n.d.). They were screened on the Prolific platform to reside in the US or UK, be fluent in the English language, have no literacy difficulties (e.g., dyslexia), have not participated in the pre-test, and have no extremely valenced opinions on vaccination (the screening question was “I believe that scheduled immunizations are safe for children: 1 totally disagree−7 totally agree;” people scoring 1 or 7 were excluded from participation. This criterion was based on the notion that people with very strong vaccine opinions might be insufficiently susceptible to a text regarding vaccination, which could suppress any potential effects of text type on availability-related variables). The 418 participants in our study were each paid £3.75 for their participation which took 24.5 minutes on average (*SD* = 9.2). For participant characteristics, see Table 1.

**Table 1:** Participant characteristics in experiment 1.

**Variable**	**Level**	***N***	**%**	***Min***	***Max***	***Mean***	***SD***
Age				18	76	44.83	12.90
Gender	Female	280	67.0				
	Male	136	32.5				
	Other	2	0.5				
Education	Elementary school	2	0.5				
	Middle school	6	1.4				
	High school	83	19.9				
	College without degree	82	19.6				
	Associate's degree	16	3.8				
	Bachelor's degree	162	38.8				
	Graduate degree	67	16.0				
Having children	Yes	236	56.5				
	No	182	43.5				
Age of parents in sample		236		24	76	47.64	12.02
Children's received vaccinations	All	207	87.7				
	Some	27	11.4				
	None	2	0.8				

### Procedure

Ethical approval was provided by the ethical committee of a large European University (file number 2019-3965). Participants were recruited and participated in December 2020. After completing informed consent procedures, participants were instructed to read a text about a social issue^1^, after which they would answer various questions about the text as well as their personal opinion on several issues.

Participants were randomly presented with one of four experimental texts. After reading the text, they first answered several questions about their demographics as well as an instructional manipulation check identifying whether participants attentively participated and followed instructions (Hauser and Schwarz, 2015). This was done to create some time between text reading and a free recall memory task. This memory task was included to facilitate the critical questions regarding ease of retrieval. Next, participants received questions measuring ease of retrieval, risk perceptions, and a manipulation check. Then, they answered questions pertaining to our exploratory variables including resistance, text evaluation, vaccination attitude, attitude certainty, personal relevance, knowledgeability, attitude source, attitude stability, preference for intuition and deliberation, and having children. Finally, participants received an open-ended question about the perceived purpose of the experiment and were carefully debriefed, referred to a government website with reliable and evidence-based information about the workings of vaccinations, and thanked for their participation.

### Stimulus Materials

Four text versions were developed that discussed early-childhood vaccination, using measles as an example. All versions were based on often-consulted sources on the internet (including official information from the vaccine-promoting website CDC.org and testimonials from the vaccine-critical website vaxtruth.org). The texts were relatively long (1,652–1,697 words) to increase the probability of participants experiencing narrative transportation (Green and Brock, 2000) and allowing differences between the texts to manifest. All texts contained general information about vaccines, as well as 12 elements describing measles symptoms and 12 elements describing vaccine side effects, each mentioned once. Effort was made to have comparable location and dispersion of these elements across conditions to account for primacy and recency effects on memory. *Content* was manipulated by (1) replacing vaccine-positive arguments from the pro-vaccine condition (e.g., about herd immunity) with vaccine-critical arguments in the anti-vaccine condition (e.g., about natural immunity) and (2) replacing the emphasis on measles symptoms in the pro-vaccine condition with an emphasis on vaccine side effects in the anti-vaccine condition. *Format* was manipulated by replacing factual contextual information from the expository text (e.g., describing the Center for Disease Control's recommendations to follow the vaccination schedule) with personal contextual information to create a narrative text (e.g., describing how a mother weighed options regarding vaccination to choose, in her specific situation, what is best for her child). This resulted in two pro-vaccine texts arguing the necessity of vaccinating to protect against vaccine preventable diseases such as measles and two anti-vaccine texts arguing the necessity of thinking critical about vaccination to protect against vaccine adverse events, either based on coherent facts (expository version) or personal experiences (narrative version). A pre-test among 20 participants (screened to have similar characteristics as the participants in experiment 1) showed that manipulations worked as intended.

### Measures

The response options for all scale questions were on a 7-point Likert scale or 7-point semantic differential, with higher scores indicating a greater extent to which the measured construct was present.

#### Dependent Variables

*Free recall*. To facilitate measurement of the availability heuristic, participants were asked to recall as much information from the text as possible. Note that for an availability heuristic to occur, it is not necessary to actually perform the operation of memory retrieval (Tversky and Kahneman, 1973). By assessing the reported memories we took into account the possibility that experienced ease of retrieval is confounded by biased recall (Iyengar, 1990) which would manifest as greater actual retrieval (Schwarz et al., 1991). Participants were encouraged to specifically report memories about vaccination and vaccine-preventable diseases in the order in which they came to mind. They had 3.5 minutes to perform this task. Participants had the option to proceed to the next question in case they found it difficult to retrieve more memories before the 3.5 minutes had passed. This option was pre-tested and based on the notion that, for an availability heuristic to occur, memory retrieval should not be perceived as too easy or difficult because this might suppress any effects.

*Ease of retrieval* (α = 0.86, *M* = 4.64, *SD* = 1.40) was assessed using three items: “the requested information came to mind easily;” “listing more arguments would have required no effort” (Ruder and Bless, 2003); “how difficult was it to recall the requested information from the text?” (Schwarz et al., 1991).

*Risk perception* was assessed using eight items. Following Ferguson and Gallagher (2007), half of the items focused on procedural risk (the risk of vaccine side effects) and half on outcome risk (the risk of vaccine preventable diseases). Following Witte (1994), the risk perception questions distinguished between susceptibility (how likely is a situation?) and severity (how serious is a situation?). Two items were derived from Betsch et al. (2010) and the other six were self-constructed. Items on *risk of vaccine side effects* (4 items, α = 0.83, *M* = 2.75, *SD* = 1.17) included “vaccinating causes considerable risks;” “how serious are the side effects of vaccines (administered against vaccine preventable diseases, such as measles)?” and on *risk of vaccine preventable diseases* (4 items, α = 0.79, *M* = 5.18, *SD* = 1.09) included “not vaccinating causes considerable risks;” “if a child is unvaccinated, how likely is it that it will get vaccine-preventable diseases (such as measles)?”.

#### Manipulation Check

To check whether the *format* manipulation worked as intended, participants were asked “to what extent did the text provide information in a narrative (personal, experience-based, story-like) manner?” and “to what extent did the text provide information in an expository (general, explanation-based, business-like) manner?” Also, transportation (α = 0.81, *M* = 5.18, *SD* = 1.09) was assessed with six items adapted from Green and Brock (2000), including “I had a vivid image of what the text was about” and “the text affected me emotionally.”

The *content* manipulation was assessed with two semantic differential items asking “in the text, how negative or positive was the sentiment toward childhood vaccines?” (−3 extremely negative—+ 3 extremely positive) and “how likely do you think it is that the author of the text would vaccinate her own children against infectious diseases?” (−3 very unlikely—+ 3 very likely).

Finally, text evaluation items asked participants to evaluate the text on several characteristics (e.g., logically ordered, boring, easy to understand).

#### Exploratory Variables

*Resistance* was divided into three constructs; cognitive resistance, affective resistance, and perceived persuasive intent. *Cognitive resistance* (α = 0.96, *M* = 3.27, *SD* = 1.87) was measured with seven items, four on counter arguing (e.g., “I found myself actively disagreeing with the author,” cf. Nabi et al., 2007) and three on negative cognitions [i.e., “the thoughts I had about this message were unfavorable; positive (reversed); bad” cf. Reynolds-Tylus et al., 2021]. *Affective resistance* (α = 0.95, *M* = 3.05, *SD* = 1.84) was measured with four items assessing anger: “while reading the message, I felt irritated; angry; annoyed; aggravated” (adapted from Dillard and Shen, 2005) and four self-created positive counterparts serving as fillers (“content; good-humored; pleased; calm”). The four items assessing anger were averaged and higher scores indicated more affective resistance. *Perceived persuasive intent* (α = 0.89, *M* = 4.46, *SD* = 1.61) was assessed with two items on perceived intent: “I believe the author wants to convince me of her point of view/tries to influence my opinions and behaviors” (based on Scherr and Müller, 2017) and two items measuring freedom threat: “I believe the text tried to pressure me/manipulate me.”

*Vaccination attitude* (α = 0.82, *M* = 5.73, *SD* = 1.28) was measured using five items adapted from Horne et al. (2015), with example items including “the risk of side effects outweighs any potential benefits of vaccines” (reverse-coded) and “if I were to make a future decision about vaccinating, I'd plan to vaccinate my child.”

*Attitude certainty* (α = 0.90, *M* = 5.73, *SD* = 1.14) was measured with three items: “how certain are you of your opinion toward vaccination?” (Tormala and Petty, 2004); “how likely are you to change your opinion about vaccination?” (reversed); “how certain are you that your opinion about vaccination is right?” (Pomerantz et al., 1995).

Other potentially relevant individual characteristics were assessed with multiple items, being *personal relevance* (“how important to you personally is the issue of vaccination?”), *knowledgeability* (“how knowledgeable do you consider yourself to be about vaccination?”), *attitude source* (“which sources have influenced your opinion about vaccination?”), *attitude stability* (“have you ever changed your opinion about childhood vaccination? If so, please explain briefly how this happened: who or what changed your mind?”). Additionally, *preference for intuition and deliberation* was assessed using eight items from Betsch and Kunz (2008), resulting in a preference for intuition scale (α = 0.74, *M* = 4.83, *SD* = 1.01) with four items (e.g., “my feelings play an important role in my decisions”) and a preference for deliberation scale (α = 0.75, *M* = 5.74, *SD* = 0.85) with four items (e.g., “before making decisions, I first think them through”). *Demographic* items inquired about gender, age, education level, first language, dominant language, having children, whether children received all / some / no vaccination, survey participation environment, and perceived purpose of the study. These variables were explored but did not systematically contribute to the most interesting explorative findings, and are therefore not reported. More information is available upon request. For all reported materials, measures, procedures, data, and syntax, see Vandeberg et al. (2022a).

## Results

### Randomization Check

Randomization checks showed that age [*F*_(3, 414)_ < 1], gender [*χ*^2^_(3)_ = 3.78, *p* = 0.29]^2^, education level [*χ*^2^_(9)_ = 4.28, *p* = 0.89]^3^, having children [*χ*^2^_(3)_ = 1.33, *p* = 0.72], and having children vaccinated [*χ*^2^_(6)_ = 4.18, *p* = 0.65] did not significantly differ across the four text conditions, which shows that randomization led to comparable distribution of participants across conditions.

### Manipulation Check

The manipulations worked as intended. Three one-way ANOVAs of format on the two perceived narrativity items and the transportation scale showed that narrative texts were indeed rated as more narrative than expository texts [*M*_*narr*_ = 6.57, *SD*_*narr*_ = 0.68; *M*_*expos*_ = 2.79, *SD*_*expos*_ = 1.73; *F*_(1, 267.45)_ = 860.18, *p* < 0.001]^4^, as less expository [*M*_*narr*_ = 3.07, *SD*_*narr*_ = 1.70; *M*_*expos*_ = 5.79, *SD*_*expos*_ = 1.22; *F*_(1, 381.10)_ = 352.85, *p* < 0.001], and resulted in greater transportation [*M*_*narr*_ = 5.54, *SD*_*narr*_ = 0.97; *M*_*expos*_ = 4.81, *SD*_*expos*_ = 1.09; *F*_(1, 416)_ = 52.32, *p* < 0.001]. Two one-way ANOVAs of content on the two perceived sentiment items showed that texts with anti-vaccination content were rated as more negative toward childhood vaccines than those with pro-vaccination content [*M*_*anti*_ = −2.07, *SD*_*anti*_ = 1.23; *M*_*pro*_ = 2.48, *SD*_*pro*_ = 0.85; *F*_(1, 373.33)_ = 1949.05, *p* < 0.001] and resulted in smaller perceived likelihood of the author of the text vaccinating their own children against infectious diseases [*M*_*anti*_ = −2.25, *SD*_*anti*_ = 1.42; *M*_*pro*_ = 2.76, *SD*_*pro*_ = 0.67; *F*_(1, 299.92)_ = 2139.80, *p* < 0.001].

### Hypothesis Testing

To test whether format (H1a) and content (RQ1a) might evoke an availability heuristic by affecting ease of retrieval, we performed a 2 (format) × 2 (content) ANOVA on ease of retrieval^5^. The results show no significant main effect of text format [*F*_(1, 414)_ = 1.30, *p* = 0.26, η^2^ = 0.00], meaning that participants experienced similar ease of retrieval for narrative and expository texts. However, there was a significant but small effect of content [*F*_(1, 414)_ = 4.95, *p* = 0.03, η^2^ = 0.01] which showed that participants reported greater ease of retrieval for pro-vaccine texts (*M* = 4.79, *SD* = 1.37) than anti-vaccine texts (*M* = 4.49, *SD* = 1.42). No significant interaction emerged (*F* < 1).

To test our hypothesis that format (H1b) and content (RQ1b) affect risk perceptions, we performed two-way ANOVAs on the risk of vaccine side effects and risk of preventable diseases. These showed a similar pattern of no significant text format effect [*F*_risk_vaccine(1, 414)_ = 1.90, *p* = 0.17, η^2^ = 0.01; *F*_risk_disease_ < 1], no significant interaction [*F*_risk_vaccine_ <1; *F*_risk_disease(1, 414)_ = 2.30, *p* = 0.13, η^2^= 0.00], but a significant and large effect of content [*F*_risk_vaccine(1, 414)_ = 69.49, *p* < 0.001, η^2^ = 0.14; *F*_risk_disease(1, 414)_ = 62.91, *p* < 0.001, η^2^ = 0.13]. The content effect shows that, compared to texts with pro-vaccination content, anti-vaccination texts increase vaccine risk perceptions (*M*_anti_ = 3.19, *SD*_anti_ = 1.28; *M*_pro_ = 2.31, *SD*_pro_ = 0.85) and decrease disease risk perceptions (*M*_anti_ = 4.79, *SD*_anti_ = 1.16; *M*_pro_ = 5.59, *SD*_pro_ = 0.83).

With no main effect of text format on ease of retrieval and risk perception, H1a and H1b are rejected: vaccine narratives do not result in greater experienced ease of retrieval and increased risk perceptions compared to expositories. The lack of significant interactions answers RQ1: pro- or anti-vaccine content does not affect any effect of narratives on (a) ease of retrieval and (b) risk perception.

### Exploratory Analyses

Various exploratory analyses were performed. The most notable findings from this set of analyses are the theoretically meaningful interaction effects presented below.

#### Cognitive Resistance

**Figure 1:**
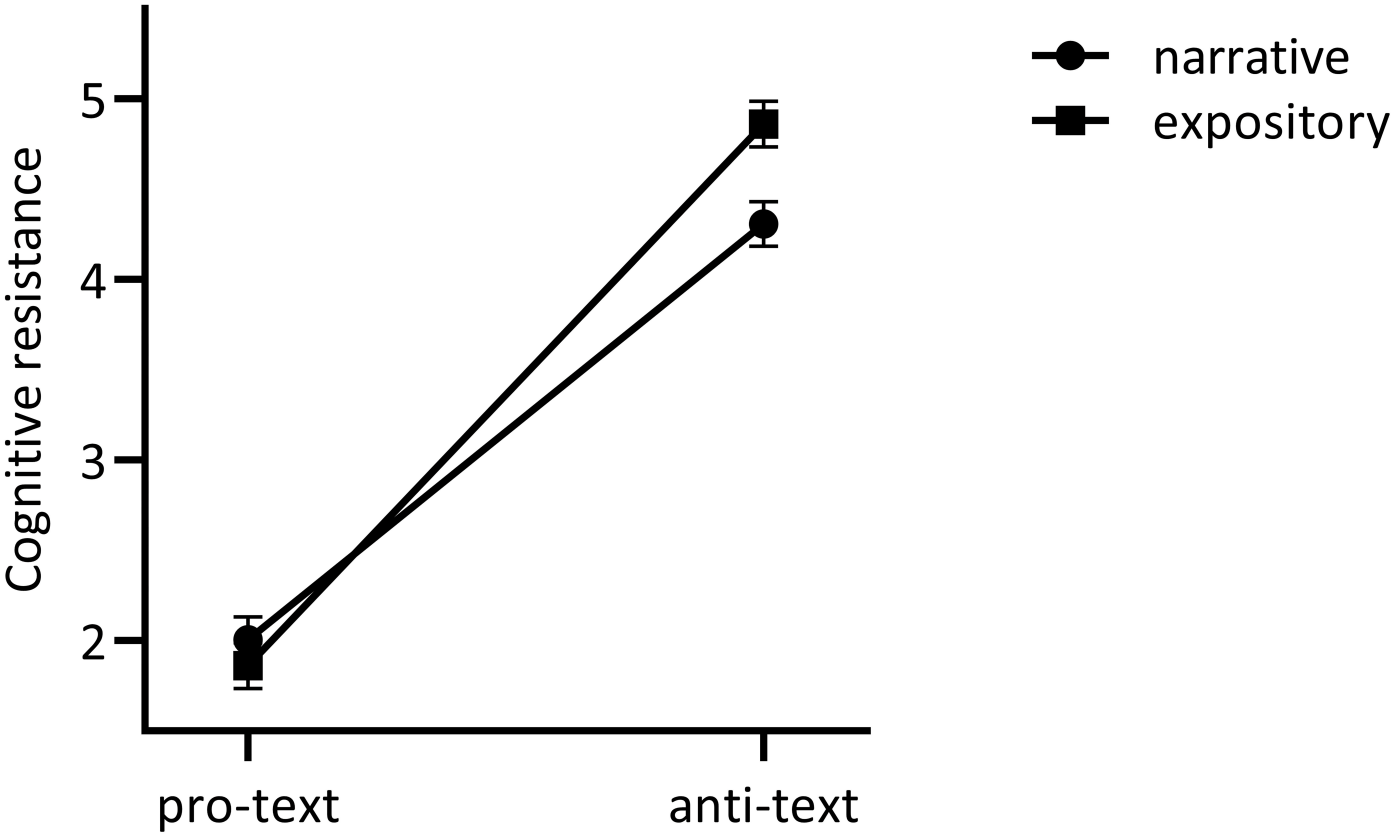
Exploratory format*content interaction on cognitive resistance. Bars reflect standard errors to the mean.

A two-way ANOVA of format and content on cognitive resistance^6^ showed no main effect of format [*F*_(1, 414)_ = 2.55, *p* = 0.11, η^2^ = 0.01], but a large effect of content [*F*_(1, 414)_ = 435.68, *p* < 0.001, η^2^ = 0.51] demonstrating more cognitive resistance toward texts with anti- (*M* = 4.58, *SD* = 1.61) vs. pro-vaccine content (*M* = 1.93, *SD* = 0.92). A significant interaction [*F*_(1, 414)_ = 7.45, *p* = 0.007, η^2^ = 0.02] showed that both pro-texts evoked equally low levels of cognitive resistance (*M*_*narr*_ = 2.00, *SD*_*narr*_ = 1.02; *M*_*expository*_ = 1.86, *SD*_*expository*_ = 0.81; *p* = 0.43), but anti-texts evoked less cognitive resistance when the text was narrative (*M* = 4.31, *SD* = 1.54) vs. expository (*M* = 4.86, *SD* = 1.63, *p* = 0.002), see Figure 1.

#### Attitude Certainty

**Figure 2:**
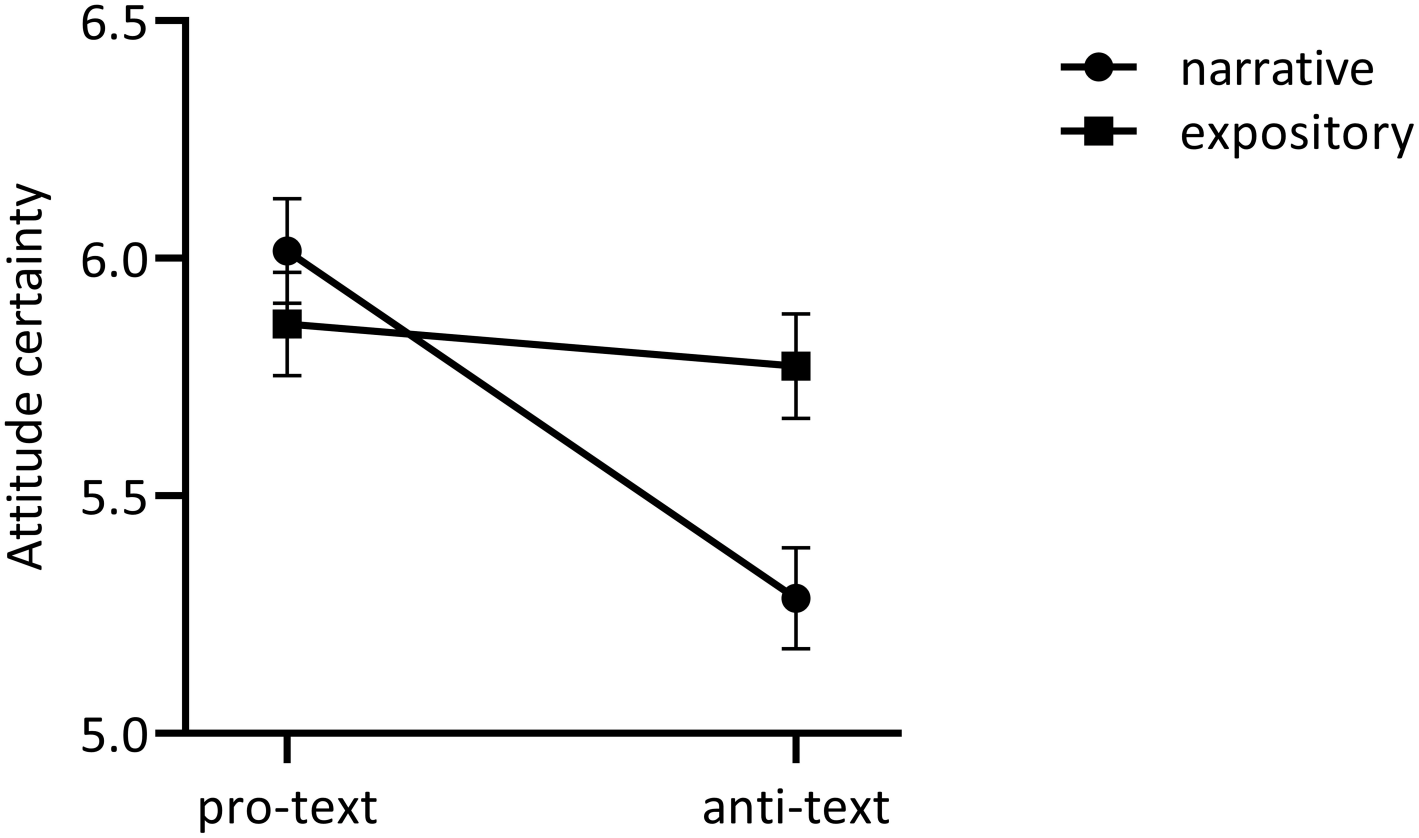
Exploratory format*content interaction on attitude certainty. Bars reflect standard errors to the mean.

A two-way ANOVA on attitude certainty^7^ showed a comparable pattern, with no main effect of format [*F*_(1, 414)_ = 1.87, *p* = 0.17, η^2^ = 0.00] and a small effect of content [*F*_(1, 414)_ = 11.49, *p* = 0.001, η^2^ = 0.03] showing that participants reading a pro-vaccine text were more certain of their attitude (*M* = 5.94, *SD* = 0.95) than those reading an anti-vaccine text (*M* = 5.52, *SD* = 1.27). The significant but small format^*^content interaction [*F*_(1, 414)_ = 7.27, *p* = 0.007, η^2^ = 0.02] showed that participants reading both pro-texts were equally certain about their vaccination attitude (*M*_*narrative*_ = 6.02, *SD*_*narrative*_ = 0.89; *M*_*expository*_ = 5.86, *SD*_*expository*_ = 1.00; *p* = 0.35), but participants reading anti-texts were more uncertain about their vaccination attitude when the text had a narrative (*M* = 5.28, SD = 1.37) vs. expository format (*M* = 5.77, SD = 1.12, *p* = 0.004), see Figure 2.

#### Moderated Moderation

**Figure 3:**
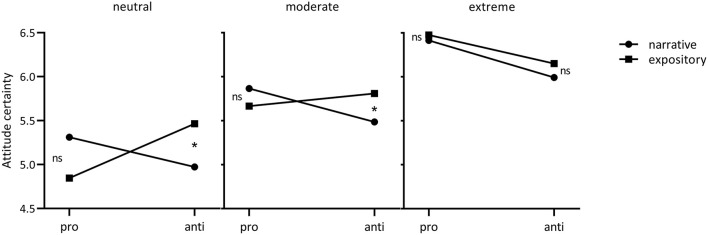
Exploratory format*content*vaccination attitude interaction on attitude certainty. The three panels reflect the extent to which participants reported a positive attitude toward vaccination, labeled “neutral” (*M* – 1 *SD*), “moderate” (*M*), “extreme” (*M* + 1 *SD*).

Given the interactions of format and content, we explored the possibility that the text effects on attitude certainty might especially hold for those people with weaker vaccination attitudes. As we had no clean measure of people's attitudes toward vaccination before reading the text, we used the assessment of vaccination attitude after reading the text as a proxy. Of course, this analysis requires cautious interpretation given the possibility that people's a priori vaccination attitudes might have potentially shifted in response to the text. We performed a moderated moderation analysis using the PROCESS macro for SPSS (model 3, Hayes, 2018). We used 5,000 bootstrap samples to estimate the 95% bias-corrected confidence intervals and we mean-centered variables^8^. The overall model with attitude certainty as dependent variable, content as independent variable, text format as moderator, and vaccination attitude as continuous moderator was significant [*R*^2^ = 0.29, *F*_(7, 410)_ = 24.66, *p* < 0.001]. Results showed no significant three-way interaction (*b* = 0.18, *t* = 1.04, BCI [−0.16; 0.51], *p* = 0.30). However, due the exploratory nature of this analysis we did further examine potential conditional effects. Conditional effects analysis of the format^*^content interaction for different values of vaccination attitude showed no significant format^*^content interaction for participants with extremely positive vaccination attitudes (+ 1 SD above the mean, *M* = 7.00, *t* = 0.69, *p* = 0.49) but did show significant interactions for those with moderately positive vaccination attitudes (*M* = 5.73, *t* = 2.03, *p* = 0.04) as well as those with more neutral (or ambivalent) attitudes around midpoint of the 7-point 5-item vaccination scale (−1 SD below the mean, *M* = 4.45, *t* = 2.10, *p* = 0.04), see Figure 3. Follow-up testing for participants with moderately positive and neutral vaccination attitudes showed that, for the pro-vaccine texts, any differences between format were non-significant (*p*s > 0.40). For the anti-vaccine texts, participants with moderately positive vaccination attitudes (*t* = 2.04, *p* = 0.04) and participants with neutral attitudes (*t* = 2.50, *p* = 0.01) showed significantly less attitude certainty after reading a narrative than an expository text. These findings suggest that people with decreasingly positive vaccination attitudes show increasingly pronounced format^*^content interaction effects, with less attitude certainty after reading an anti-vaccine narrative vs. expository. This suggests that anti-vaccination narratives might mainly impact the attitude certainty of people with relatively neutral (vs. extremely positive) vaccination attitudes.

## Discussion

Overall, the findings show no empirical support for a role of the availability heuristic in response to vaccination information. That is, reading a narrative text about vaccination did not result in greater experienced ease of retrieval (H1a) and increased risk perceptions (H1b) than reading an expository text, even when taking the anti- vs. pro-vaccine content of the text into account (RQ1). These findings are not in line with the theoretical assumptions made in the literature about the effect of narratives on the availability of information in memory (McGregor and Holmes, 1999) and subsequent risk perceptions (e.g., Serpell and Green, 2006; Kuru et al., 2021), nor with the notion that especially information about vaccine adverse events is memorable (Miton and Mercier, 2015; Omer et al., 2017). Our findings by no means disqualify these theoretical assumptions and correlational interpretations, but they do stress the necessity to narrow these assumptions down into concrete, testable predictions regarding the causal role of the availability heuristic. This heuristic might still play a role in the process leading up to a vaccine decision in real life, but the part of the process that was highlighted in our current test found no evidence for such a role. Specifically, our findings suggest that the availability heuristic does not play a *causal* role in the *short-term* effects of *processing information* about vaccines.

With regards to the experimental paradigm, classical experiments demonstrating the availability heuristic usually ask people to retrieve multiple instances from memory based on long-term personal experiences, for instance semantic memory regarding the number of words with the letter R in the first vs. third position (Tversky and Kahneman, 1973) or episodic memory regarding people's personal past assertive or unassertive behavior (Schwarz et al., 1991), after which participants have to produce estimates based on those instances. Our study asked people to retrieve information from memory based on short-term experiences with one text they had just read in an experimental set-up, before producing estimates. It might be the case that an availability heuristic only manifests when memories are long-term, generalizable (i.e., consist of various instances), accumulated, or more grounded in personally relevant, real-life experiences.

A related but different point is that we assessed people's ease of retrieval and risk perceptions shortly after reading the text, whereas potential availability effects relating to a specific piece of information might manifest over a longer time span. Indeed, from the perspective of narrative effect research, this is a viable option, as various lines of research show that narratives might mainly have effects on the longer-term (McGregor and Holmes, 1999; Appel and Richter, 2007; Moyer-Gusé et al., 2011), which we may have missed in the current paradigm testing short term effects.

Regarding our sample, the participants reported generally positive vaccination attitudes (*M* = 5.73, *SD* = 1.28, *Median* = 6, *Mode* = 7). This indicates that our overall sample likely had relatively positive pre-existing beliefs regarding the topic of vaccination, potentially leaving insufficient room for a single forced exposure to a text to reliably alter people's memories of a vaccine-related text and existing risk perceptions. This possibility can be tested in future research, by contrasting peoples' responses to texts about topics for which they do not (vs. do) have pre-existing beliefs or knowledge. However, if an availability heuristic cannot occur in the presence of prior knowledge or beliefs regarding a topic, it is virtually impossible to assess the causal role of this heuristic in the domain of vaccination, as most people will have pre-existing knowledge and beliefs regarding vaccines. Though this complicates the assessment of the availability heuristic, future work should address this given the weight that this heuristic receives in the literature on vaccine decisions.

Interestingly, the dependent variables addressing the availability heuristic did reveal a non-hypothesized main effect of content, showing that pro- (vs. anti-) vaccine texts resulted in greater perceived ease of retrieval, decreased vaccine risk perceptions, and increased disease risk perceptions. This unexpected finding shows that our measures were sensitive to variations in the text. Perhaps, the generally vaccine-positive sample found it easier to recall pro-vaccination information. This would be in line with schema theory and cognitive psychological research evidencing that it is easier to interpret and store new information if it can be associated with existing knowledge in long-term memory (cf. Anderson and Pearson, 1984). Alternatively, the affected ease of retrieval and risk perception measures might be a manifestation of the generally vaccine-positive people strengthening their existing beliefs when reading a pro-vaccine text. In this case, the current data might reflect a confirmation bias, which is “a general tendency for people to believe too much in their favored hypothesis” (Klayman, 1995). Research shows that the confirmation bias indeed affects the processing of information about vaccines (Meppelink et al., 2019). Though confirmation bias is indeed theorized to interact with the availability heuristic (Sunstein, 2006), future research should further explore this relation and identify whether the current effects were a manifestation of the availability heuristic that serves as an antecedent or consequence of confirmation bias, or a result of confirmation bias itself.

Finally, the exploratory findings interestingly suggest that especially anti- (vs. pro-) vaccination narratives increase cognitive resistance and reduce attitude certainty. When people read an anti-vaccine *narrative* they reported less cognitive resistance and less attitude certainty than when they read an anti-vaccine *expository*. Furthermore, the additional conditional effects analysis suggests that anti-vaccination narratives might mainly impact the attitude certainty of people with relatively neutral (vs. extremely positive) vaccination attitudes. However, given that the current vaccination attitude measure may reflect a combination of both a priori vaccination attitude and a potentially shifted post-reading vaccination attitude, a confirmatory experiment will have to distinguish between the two constructs to test the hypotheses derived from the exploratory findings. Hence, we performed a preregistered follow-up experiment to examine whether (1) anti-vaccination narratives are indeed more persuasive than anti-vaccination expositories; (2) this is caused by less cognitive resistance when reading narrative texts; (3) a persuasive effect of anti-vaccination narratives is stronger as people are a priori more neutral or hesitant (vs. vaccine-positive).

## Experiment 2

As outlined above, especially anti-vaccine narratives are argued to affect people's perceptions regarding childhood vaccines. Though experiment 1 showed no effects of an anti-vaccine narrative (vs. expository) on the assessment of the availability heuristic, it did show exploratory effects on cognitive resistance and attitude certainty. This is in line with recent evidence indicating that weak facts (which can be roughly compared to non-scientific anti-vaccine arguments) are more persuasive when presented in stories than when presented in isolation (Krause and Rucker, 2020). It is also in line with ample evidence from various fields showing that narratives in different forms can reduce resistance (e.g., Moyer-Gusé and Nabi, 2010; Niederdeppe et al., 2011) and—through various mechanisms—result in story-consistent judgments (e.g., McGregor and Holmes, 1999) and attitudes (e.g., de Graaf et al., 2012). A recent meta-analysis presents convincing evidence that narratives indeed generate less resistance than non-narratives (Ratcliff and Sun, 2020). Support for the Entertainment Overcoming Resistance Model (Moyer-Gusé, 2008) further shows that narrative structures can reduce resistance which, in turn, stimulates text-consistent attitudes and behaviors (Moyer-Gusé et al., 2011). Combining this literature with the findings of experiment 1, we hypothesize:

*H2. An anti-vaccine narrative will result in a) less cognitive resistance and b) more negative vaccination attitudes than an anti-vaccine expository*.
*H3. Cognitive resistance mediates the effect of text format on vaccination attitudes. That is, reading an anti-vaccine narrative (vs. expository) will result in reduced cognitive resistance, which will in turn result in more negative vaccination attitudes*.

Furthermore, the exploratory findings of experiment 1 suggest that the persuasive effect of anti-vaccine narratives might particularly hold for people with relatively neutral vaccination attitudes, compared to those with extremely positive attitudes. Though the concept of vaccine hesitancy has been used heterogeneously and encompasses a range of attitudes and behaviors (Dubé et al., 2016a), people with attitudes between both ends of the vaccine attitude continuum are considered vaccine-hesitant (Dubé et al., 2016b). Based on this reasoning, our findings can indicate that especially vaccine-hesitant individuals might be affected by anti-vaccine narratives, whereas vaccine-positive individuals might be less susceptible to these effects.

This is in line with literature stating that it is very difficult to change beliefs once they are formed (Slovic, 1986; Pluviano et al., 2017). Evidence shows that pre-existing (accurate and inaccurate) vaccination beliefs indeed stably predict their post-intervention counterparts, which demonstrates belief consistency effects (Kessler et al., 2019). Further evidence shows that attitude ambivalence and attitude certainty predict attitude stability over time, showing that less valenced and less certain attitudes are less enduring and less resistant to change (Luttrell et al., 2016). Extending these findings to vaccine hesitancy, it is argued that people whose vaccine attitudes are relatively ambivalent or weak are likely more susceptible to persuasive claims (Stasiuk et al., 2021). Finally, especially people who lack strong prior opinions are vulnerable to the format in which information is presented (Slovic, 1986). We therefore hypothesize:

*H4. Prior vaccine hesitancy moderates the effect of text format on vaccination attitudes. That is, the effect of an anti-vaccination narrative (vs*. *expository) on vaccination attitudes will be stronger for people who are a priori more hesitant*.

The dependent variable in the hypotheses is formulated in terms of vaccination attitudes, even though the exploratory findings showed effects on attitude certainty. The reason for this is three-fold. First, empirical findings demonstrate a vital role for vaccination attitude in predicting vaccination intentions (Paulussen et al., 2006; Xiao and Wong, 2020), stressing the importance of vaccination attitudes in vaccine decisions. Second, attitudes consist of various dimensions, including valence/ambivalence (how positive and/or negative attitudes are), strength (how strong attitudes are), and certainty (how certain people are of their attitude). Given that the attitude measure in experiment 1 provided no “clean” measure of pre- or post-reading vaccine attitudes, we reasoned that attitude certainty provided the best proxy for potential attitude shifts in experiment 1. However, with the improved design of experiment 2, we were able to put more focus on the most-often assessed dimension of attitude, being valence. Third, given that valence and certainty are likely to complement each other (with people being very certain of their highly positive or negative attitudes and not so certain of their relatively neutral attitudes) but might also contradict (with people being not so certain of their highly positive or negative attitudes and being very certain of their relatively neutral attitudes), we added both dependent variables to our experiment, extending H4 into the following research question:

*RQ2. Is there an interaction effect between vaccine hesitancy and text format on attitude certainty?*

**Figure 4:**
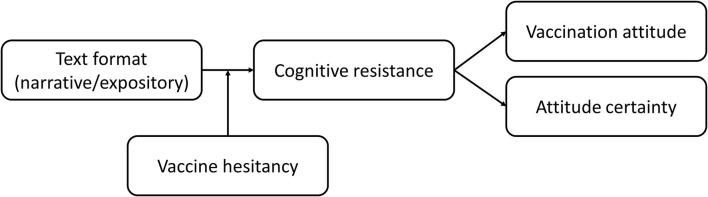
Conceptual model.

This reasoning results in the conceptual model depicted in Figure 4.

Testing the entire model results in the following research question:

*RQ3. Does cognitive resistance mediate a vaccine hesitancy* ^*^ *text format interaction on a) vaccination attitudes and b) attitude certainty?*

## Materials and Methods

### Design and Participants

Based on the findings of experiment 1, we focused on the texts with anti-vaccination content and adopted a one factorial (text format: narrative/expository) between-subjects design with three dependent variables (cognitive resistance, vaccination attitude, attitude certainty). The moderator (vaccine hesitancy) was measured 1–6 days prior to the experiment. Participants were recruited through Prolific Academic and screened on characteristics identical to experiment 1 (including no participation in the prior experiment). However, to ensure a more equal distribution of participants, ranging from vaccine-hesitant to extremely positive, we specifically recruited over 200 participants who reported moderate vaccine opinions in a screening question (scoring 3, 4, 5 on the 7 point scale) as well as over 200 participants with more positive opinions (scoring up to 7).

Based on a power analysis (see preregistration), we recruited minimally 400 participants, which corresponds to a pre-calculated power of almost 0.85. A total of 445 participants completed both parts of the study. Forty-two participants were discarded from the dataset because they spent an insufficient amount of time on the text page to ensure attentive reading of the text as instructed (<50 s, identified as outliers in the histogram plotting time on page) *and* failed either an instructional manipulation check (Hauser and Schwarz, 2015) and/or an attention check regarding content of the text. This is a slight deviation from the preregistered data exclusion criteria, informed by unforeseen practical limitations of the prior criteria.

The remaining 403 participants were each paid £2,75 for their participation in both parts of the online experiment (£0.65 for part 1 with a mean duration of 4.8 min and £2.10 for part 2 with a mean duration of 13.9 min). See Table 2 for participant characteristics.

**Table 2:** Participant characteristics in experiment 2.

**Variable**	**Level**	***N***	**%**	***Min***	***Max***	***Mean***	***SD***
Age				18	76	38.21	13.28
Gender	Female	283	70.2				
	Male	115	28.5				
	Other	5	1.2				
Education	Elementary school	1	0.2				
	Middle school	6	1.5				
	High school	89	22.1				
	College without degree	111	27.5				
	Associate's degree	18	4.5				
	Bachelor's degree	128	31.8				
	Graduate degree	50	12.4				
Having children	Yes	193	47.9				
	No	210	52.1				
Age of parents in sample		193		21	76	44.64	11.64
Children's received vaccinations	All	158	81.9				
	Some	32	16.6				
	Ambiguous about whether children were vaccinated	3	1.6				

### Procedure

Ethical approval was provided by the ethical committee of a large European University (file number 2019-3965). The procedure was identical to that in experiment 1, with some exceptions detailed here. The experiment was preregistered at the Open Science Framework (Vandeberg et al., 2022b). Data collection occurred from late April to early June 2021. Participants were recruited to participate in a two-part study, with at least 24 h between part 1 and part 2. In part 1, participants were instructed to complete a survey including the critical vaccine hesitancy measure, as well as filler scales assessing, for example news media skepticism and financial beliefs, and concluded with demographic questions. The instructions and filler questions were designed to conceal our focus on vaccine hesitancy, to minimize the chances of obtaining consistency effects.

Twenty-four hours after completing part 1, participants received an invitation for part 2 to complete within the next 5 days. In part 2, participants were randomly presented with one of two texts. After reading the text, they sequentially received questions pertaining to demographics, an instructional manipulation check, vaccination attitude, attitude certainty, cognitive resistance, perceived purpose of the experiment, manipulation and attention check, having children, and an exploratory variable on whether and how the recent COVID-19 pandemic had changed their views of vaccination. Finally, they were carefully debriefed, thanked, and referred to a government website with reliable and evidence-based information about the workings of vaccinations.

### Stimulus Materials and Measures

The anti-vaccine texts from experiment 1 were used (see Appendix X). The dependent variables (cognitive resistance, vaccination attitude, and attitude certainty), demographic questions, and manipulation and attention checks were assessed as in experiment 1.

*Vaccine hesitancy* was measured 1–6 days prior to experimental exposure to the text. It was assessed using the same items that were also used to measure vaccine attitudes in experiments 1 and 2 (Horne et al., 2015), but with a different response scale to minimize potential consistency effects. For these 5 items, the 7-point scale was replaced with a slider ranging from −50 (strongly disagree) to 50 (strongly agree). Following the conceptualization from earlier work (Dubé et al., 2016b), we conceptualize individuals with seemingly neutral attitudes (around scale midpoint, e.g., between −25 and 25) as more vaccine-hesitant than individuals with relatively positive attitudes (i.e., between 25 and 50). The scale's reliability was acceptable (α = 0.73) and revealed heterogeneous vaccine hesitancy scores (*M* = 24.69, *SD* = 18.37, with 50% of the sample scoring below 25.50 and 50% scoring above). Again, see Vandeberg et al. (2022a) for all materials pertaining to the methods and results.

## Results

### Randomization Check

Randomization checks showed that age [*F*_(1, 401)_ < 1], gender [*χ*^2^_(1)_ = 0.98, *p* = 0.32]^9^, education level [*χ*^2^_(4)_ = 6.31, *p* = 0.18]^10^, having children [*χ*^2^_(1)_ = 0.62, *p* = 0.43], and having children vaccinated [*χ*^2^_(1)_ = 0.11, *p* = 0.74]^11^ did not significantly differ across the two text conditions.

### Manipulation Check

The manipulation worked as intended. Two one-way ANOVAs of format showed that narrative texts were indeed rated as more narrative than expository texts [*M*_*narr*_ = 6.41, *SD*_*narr*_ = 0.96; *M*_*expos*_ = 2.91, *SD*_*expos*_ = 1.62; *F*_(1, 291.89)_ = 669.44, *p* < 0.001]^12^ and as less expository [*M*_*narr*_ = 3.01, *SD*_*narr*_ = 1.56; *M*_*expos*_ = 5.49, *SD*_*expos*_ = 1.18; *F*_(1, 394.76)_ = 329.17, *p* < 0.001].

### Hypothesis Testing

**Figure 5:**
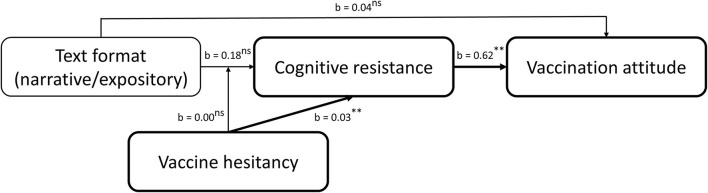
Moderated mediation on vaccination attitudes. ns, non-significant; **p < 0.001.

We analyzed the data using the PROCESS macro for SPSS (Hayes, 2018). We used 50,000 bootstrap samples to estimate the 95% bias-corrected bootstrap confidence intervals (BCIs) and used heteroscedasticity-consistent standard errors to account for violation of the homoscedasticity assumption by the cognitive resistance variable^13^. To test the overall model to answer H2 and RQ3, we performed mediated moderation analyses using model 7 with mean-centered products, including format (narrative vs. expository) as independent variable, vaccine hesitancy as continuous moderator, cognitive resistance as mediator and either vaccination attitudes or attitude certainty as dependent variable. See Figure 5 for an overview of the findings. Text format had no significant effect on cognitive resistance (*b* = −0.18, *t* = −1.50, 95% CI [−0.42; 0.06], *p* = 0.14). This shows that the anti-vaccine narrative did not result in less cognitive resistance than the anti-vaccine expository, thereby rejecting H2a. Interestingly, vaccine hesitancy did significantly predict cognitive resistance (*b* = 0.03, *t* = 9.77, 95% CI [0.03; 0.04], *p* < 0.001). As a higher score on the hesitancy scale indicates a more positive attitude (i.e., less hesitancy), the positive unstandardized *b*-value shows that individuals with more positive prior vaccine attitudes show more cognitive resistance against the anti-vaccine text. Thus, more hesitant individuals report less cognitive resistance against the anti-vaccine text. The text format^*^vaccine hesitancy interaction effect on cognitive resistance was non-significant (*b* = 0.00, *t* = −0.49, 95% CI [−0.02; 0.01], *p* = 0.62). Text format did not have a significant direct effect on vaccine attitudes (*b* = 0.04, *t* = 0.35, 95% CI [−0.17; 0.24], *p* = 0.72). As the anti-vaccine narrative did not result in more negative attitudes than the anti-vaccine expository, we reject H2b. However, cognitive resistance did significantly and positively predict vaccination attitudes (*b* = 0.62, *t* = 15.58, 95% CI [0.54; 0.70], *p* < 0.001). The index of moderated mediation was non-significant (index = −0.00, boot SE = 0.00, BCI [−0.01; 0.01]), thereby answering RQ3a.

**Figure 6:**
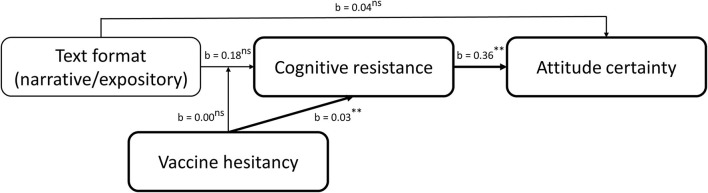
Moderated mediation on attitude certainty. ns, non-significant; **p < 0.001.

Next, the same analysis was performed with attitude certainty as dependent variable, see Figure 6. The model showed that cognitive resistance significantly predicts attitude certainty (*b* = 0.36, *t* = 8.17, 95% CI [0.27; 0.45], *p* < 0.001), showing that more cognitive resistance against the anti-vaccine text was associated with greater attitude certainty. The direct effect of text format on attitude certainty was non-significant (*b* = −0.04, *t* = −0.40, 95% CI [−0.26; 0.17], *p* = 0.69). Also, the index of moderated mediation was non-significant (index = −0.00, boot SE = 0.00, BCI [−0.01; 0.00]), thereby answering RQ3b.

Then, to test H4 and RQ2 regarding the text format^*^vaccine hesitancy interactions, we tested model 1^14^ with text format as mean-centered independent variable, vaccine hesitancy as mean-centered moderator, and either vaccination attitude or attitude certainty as dependent variable. The format^*^vaccine hesitancy interaction on vaccine attitudes was non-significant (*b* = 0.00, *t* = −0.30, 95% CI [−0.01; 0.01], *p* = 0.77). Because prior vaccine hesitancy does not moderate any effect of format on vaccination attitudes, we reject H4. Also, the text format^*^vaccine hesitancy interaction effect on attitude certainty was non-significant (*b* = 0.01, *t* = 0.89, 95% CI [−0.01; 0.02], *p* = 0.37), thereby answering RQ2.

Finally, to test H3 regarding a mediating role for cognitive resistance in the relation between text format and vaccination attitudes, we tested model 4 with 50,000 bias-corrected bootstrapped samples. The total effect of the model with text format as independent variable, cognitive resistance as mediator and vaccination attitude as dependent variable was non-significant [*R*^2^ = 0.00, *F*_(1, 401)_ < 1], as were the direct effect (*b* = 0.04, *t* = 0.35, *p* = 0.72) and indirect effect (*b* = −0.14, *boot SE* = 0.08, *BCI* [−0.31; 0.02]). As these results show no evidence for a mediation, H3 is rejected.

In sum, these findings show that text format has no effect on cognitive resistance (rejecting H2a) and vaccination attitudes (rejecting H2b), that cognitive resistance does not mediate an effect of text format on vaccination attitudes (rejecting H3), that vaccine hesitancy does not moderate an effect of text format on vaccination attitudes (rejecting H4), and that the overall proposed moderated mediation on vaccine attitudes does not hold (thereby answering RQ3a). Also, vaccine hesitancy does not moderate an effect of text format on attitude certainty (answering RQ2) and the overall proposed moderated mediation on attitude certainty is not significant (answering RQ3b).

Interestingly, the results do however show that prior vaccine hesitancy predicts cognitive resistance, and that cognitive resistance predicts vaccination attitudes and attitude certainty. To explore whether this results in a significant mediation, we tested an additional model 4 with 50,000 bias-corrected bootstrapped samples. The total effect of the model with vaccine hesitancy as independent variable, cognitive resistance as mediator and vaccination attitude as dependent variable was significant [*R*^2^ = 0.47, *F*_(1, 401)_ = 329.16, *p* < 0.001], as were the direct effect (*b* = 0.04, *t* = 12.29, *p* < 0.001) and the indirect effect (*b* = 0.01, *boot SE* = 0.00, *BCI* [0.01; 0.02]). Similarly, the same model with attitude certainty as dependent variable showed a significant total [*R*^2^ = 0.40, *F*_(1, 401)_ = 70.55, *p* < 0.001], direct (*b* = 0.02, *t* = 5.03, *p* < 0.001) and indirect (*b* = 0.01, *boot SE* = 0.00, *BCI* [0.01; 0.01]) effect. These mediation outcomes show that individuals who are a priori more vaccine-hesitant show less cognitive resistance when reading an anti-vaccine text, and in turn show less positive vaccination attitudes and less attitude certainty.

## Discussion

Experiment 2 shows that an anti-vaccination text in different formats does not differentially affect cognitive resistance, vaccination attitudes, and attitude certainty. Similarly, experiment 1 shows that a narrative format is not more likely to affect ease of retrieval and risk perceptions than an expository format. This is not in line with the literature stressing the persuasive nature of narratives, but rather contributes to the mixed findings on the effectiveness of vaccination narratives.

To interpret our findings, we first zoom out on the narrative persuasion literature as a whole. Experimental research on the impact of narratives about health-related topics is quite heterogeneous (cf., Graaf et al., 2016) in terms of the types of narratives that are used as well as the control conditions to which these are contrasted. A narrative format, for instance, is often compared to various formats containing statistical information (Allen and Preiss, 1997; Zebregs et al., 2015). However, these two conditions often differ in many ways, such as the order in which information is presented, visuals, tone-of-voice, to name a few. In our current work, we have put great effort into creating narrative and expository texts that were both as comparable and ecologically valid as possible. The described disease symptoms and side effects were identical in both versions, as well as the dispersion of elements throughout the text, overall structure (pro- or anti-vaccine) arguments, visuals, text length, and overall conclusion. This way, we aimed for a clean and stringent test of the effectiveness of a core feature distinguishing narratives from other text formats; personal experiences. Though this worked as intended, as illustrated by the manipulation checks, no differences in narrative impact were revealed. This suggests that the large number of choices that are made in the construction of narratives (for some examples see Braddock and Dillard, 2016; Graaf et al., 2016) as well as the format that narratives are contrasted with might make or break any persuasive narrative effects.

Zooming back in on the studies that have found advantageous effects of a narrative format when communicating about vaccination, we compare those designs to ours as this might indicate under which circumstances vaccine narratives are effective. For instance, Betsch and colleagues (e.g., Betsch et al., 2011, 2015; Haase et al., 2020) provide ample evidence that parents' risk perceptions and intentions to vaccinate their children against a hypothetical disease decline, as the relative frequency of narratives reporting vaccine adverse events increase. Comparing these findings to ours shows several possibilities. One very viable possibility is that pre-existing knowledge, experiences, attitudes, or beliefs determine how susceptible people are to narrative persuasion. The cited studies have assessed the perceptions of vaccines combatting *hypothetical* diseases, which indicates that narrative effects might mainly occur when people lack prior knowledge, beliefs, or attitudes regarding the specific vaccine-preventable disease that is mentioned in the text. Although we have taken a first step to address this possibility in experiment 2 by taking prior attitudes into account in the operationalization of vaccine hesitancy, our findings show that a more hesitant stance does not make people more prone to persuasion by narratives. However, even the vaccine-hesitant people in our sample likely have ample prior knowledge about and experiences with childhood vaccinations. Thus, perhaps narrative persuasion mainly occurs in the absence of prior experiences with a specific vaccine and disease. This would make a narrative format a less effective tool in the current attempt to effectively provide people with evidence-based information about existing childhood vaccines.

A different but related possibility is that the relative amount of presented (pro- or anti-) vaccination narratives—or the amount of presented experiences within a narrative—determines how susceptible people are to narrative persuasion. Possibly, narrative effects only occur as described experiences with vaccines accumulate, or in other words, anecdotal narrative evidence might only begin to receive weight as more and more evidence is presented. In our experiments, people were exposed to one single-case narrative in a single exposure. Combining our findings with those cited (e.g., Betsch et al., 2011, 2015; Haase et al., 2020) suggests that perhaps narratives are mainly effective when multiple narratives are presented describing various experiences with vaccines. Combining the different points made, the amount of narrative evidence that is needed to have an impact likely depends on the extent to which people have prior experiences with the topic at hand.

Another explanation for our findings is that narratives might mainly elicit affective mechanisms (Wroe et al., 2005; Dunlop et al., 2008; Betsch et al., 2010; Oliver et al., 2012; Sprengholz and Betsch, 2020) rather than cognitive mechanisms (cf. Miton and Mercier, 2015). This finding would be in line with the conclusion by Zebregs et al. (2015) that narrative evidence mainly affects intentions through an affective route, whereas statistical evidence mainly affects beliefs and attitudes through a cognitive route. This is roughly in line with a dual process line of reasoning (cf. e.g., Chen and Chaiken, 1999; Slovic et al., 2004; Kahneman, 2011) by suggesting that formats characterized by personal experiences are likely to elicit more intuitive, fast, automatic processes whereas formats presenting impersonal facts would likely evoke more reflective, effortful, elaborative processes. However, this line of reasoning also suggests that the intuitive processes elicited by narratives are likely to result in the use of heuristics, which we cannot confirm based on our findings from experiment 1. Therefore, this possibility requires further examination in which affective vs. cognitive or intuitive vs. reflective processes are assessed within one experimental paradigm.

Specifically, experiment 2 shows that pre-existing vaccine hesitancy (but not narrative format) predicts cognitive resistance and post-reading vaccination attitudes and attitude certainty. That is, people who are more hesitant create less counterarguments and experience less negative cognitions when reading an anti-vaccination text, and consequently report less positive attitudes and are less certain about these attitudes. This demonstrates belief consistency effects, rather than narrative persuasion effects. This is in line with findings by Kessler et al. (2019) as well as with our earlier observation that prior knowledge, experiences, beliefs and attitudes weigh heavily on the way in which people process, retrieve and perceive information regarding vaccines. Belief consistency could have suppressed potential effects of text format in our study. Research shows that reasons for vaccine belief consistency effects can be attitude bolstering, cognitive consistency, and a preference for complete mental models—even when these are (partly) inaccurate (Pluviano et al., 2017). Our findings show that it is interesting to further examine such (cognitive) mechanisms underlying belief consistency effects to gain further insights into how these can be minimized.

### Limitations

Despite our efforts, this research has its limitations. To provide a clean and rigid empirical test of the cognitive mechanisms that might be evoked by a narrative format, we presented participants with well-balanced texts in a single, forced exposure between subjects. However, presenting people with multiple narratives or multiple exposures would have been more likely to elicit narrative effects (cf. Haase et al., 2020), especially because pre-reading attitudes seemed so persistent (cf. Pluviano et al., 2017). This could be done in future research by presenting people with multiple exposures to single-case narratives or single exposure to multiple-case narratives about real-life vaccines and vaccine preventable diseases. Also, the forced exposure to the texts in our experiments was not optimally ecologically valid. Future work might try to present information through voluntary exposure (i.e., when parents have searched for or selected the information themselves, preferably when motivated to do so), though voluntary exposure also has its practical and ecological limitations in an experimental setting.

Additionally, although text conditions in the current experiments were well-controlled and ecologically valid, they may not have been optimally distinctive. Not only the expository versions, also the narrative versions can be considered as argumentative to some extent. That is, the narratives did not only *show* what conclusion should be drawn from narrative events, but they also *argued* for the presented (pro- or anti-vaccine) message, particularly since the narrative character explicitly shares reasons for this point of view based on her experiences. Narratives with explicit reasoning may be more comprehensible for some target groups (de Graaf et al., 2017), but may also increase the perceived subjectivity. Future work might distinguish between narratives with a showing, *demonstrative* style (primarily providing access to observable narrative events) vs. a more telling, *invasive* style (providing additional access to the inner and spoken reasons and evaluations of people in the narrative) (cf. van Krieken and Sanders, 2021). A detached, demonstrative style might increase perceived objectivity and therefore be more acceptable for a critical audience [as hypothesized by Sanders and van Krieken (2019)].

## Conclusion

Our two experiments show that vaccination narratives (vs. well-balanced expositories) do not result in (a) greater ease of retrieval, (b) increased risk perceptions, (c) decreased cognitive resistance, and (d) changes in vaccination attitudes or attitude certainty. This does however not rule out the possibility that text format affects the elicitation of an availability heuristic or persuasion through cognitive resistance. The most parsimonious conclusion is that, in the current set-up, these cognitive responses were outweighed by belief consistency processes, which demonstrably affected the way in which people processed information as well as their post-reading vaccine perceptions. This stresses the necessity of taking prior knowledge, experiences, beliefs, and attitudes into account when formally studying the impact of communication on highly debated topics like real-life vaccines. This rationale especially holds now that the current COVID-19 pandemic has made vaccination such a salient, omnipresent, and pressing topic, which has arguably also affected people's risk perceptions and hesitancy regarding routine childhood vaccinations (He et al., 2022). The discussion highlights potentially fruitful ways in which science should further examine whether, how, and to what extent strategic communication has the potential to change pre-existing beliefs. An important implication for stakeholders such as healthcare providers, communication specialists, and policy makers is that they should not blindly trust in storytelling techniques as the solution for current (mis-)perceptions. However, combining our findings with the literature does suggest that vaccine risk communication in a narrative format might help reach affective objectives, especially when people with more experiences and stronger prior vaccine attitudes are exposed to more instances of narrative evidence. Nonetheless, our findings show that a narrative format is not necessarily a more effective way to provide evidence-based information than the more frequently used expository format, as narrative impact is likely context-dependent and relies on many factors that should be further investigated.

## Data Availability Statement

The datasets presented in this study can be found in online repositories. The names of the repository/repositories and accession number(s) can be found at: Open Science Framework: https://osf.io/ygxmt/.

## Ethics Statement

The studies involving human participants were reviewed and approved by the Ethics Assessment Committee Humanities (EACH), Radboud University, Nijmegen, the Netherlands (file number 2019-3965). The patients/participants provided their written informed consent to participate in this study.

## Author Contributions

LV, MF, CM, and JS: conceptualization, funding acquisition, methodology, resources, and writing. LV: data curation, investigation, project administration, software, supervision, and validation. LV, CM, and MF: formal analysis. All authors contributed to the article and approved the submitted version.

## Funding

This work was supported by funding from the Centre for Language Studies and the Behavioral Science Institute of Radboud University, Nijmegen, as well as the Amsterdam School of Communication Research, University of Amsterdam, Netherlands.

## Conflict of Interest

The authors declare that the research was conducted in the absence of any commercial or financial relationships that could be construed as a potential conflict of interest.

## Publisher's Note

All claims expressed in this article are solely those of the authors and do not necessarily represent those of their affiliated organizations, or those of the publisher, the editors and the reviewers. Any product that may be evaluated in this article, or claim that may be made by its manufacturer, is not guaranteed or endorsed by the publisher.

## Footnotes

^1^We used general wording to avoid recruiting only people with a particular interest in vaccination.

^2^Excluding the non-binary individuals from this check as Chi square testing requires cell counts ≥5.

^3^Grouping individuals completing elementary, middle, and high school together and individuals completing an associate's degree and a bachelor's degree together to prevent cell counts < 5.

^4^Because the data of all manipulation check variables except the transportation mean are heteroscedastic and non-normally distributed, Welch's F-statistics are reported (cf. Delacre et al., 2019).

^5^Risk of vaccine side effects was right skewed and therefore log transformed; risk of preventable diseases was left skewed and therefore reversed (maximum score + 1 – value) before log transformation. The transformed variables were homoscedastic and normally distributed. In this and the following analyses, whenever data were transformed, the reported test statistics are based on the transformed variables; the reported descriptive statistics are non-transformed to facilitate interpretation.

^6^The cognitive resistance data are platykurtic (kurtosis = −1.11) and heteroscedastic. Because there is no easy solution to deal with the violations of both assumptions in a two-way ANOVA, the probability of a Type 1 error may be inflated and therefore outcomes should be interpreted cautiously.

^7^Attitude certainty was left skewed and reversed before log transformation, resulting in homoscedastic data with a normal distribution.

^8^Like vaccination attitudes, attitude certainty was left skewed and therefore reversed and log transformed. This resulted in homoscedastic data with a normal distribution. There was no evidence for multicollinearity between the two variables.

^9^Excluding the non-binary individuals from this check as Chi square testing requires cell counts ≥5.

^10^Grouping individuals completing elementary, middle, and high school as highest education level together to prevent cell counts < 5.

^11^Excluding the 3 unclear accounts from this check to prevent cell counts < 5.

^12^Reporting Welch's F for both manipulation checks.

^13^All other assumptions were met for all variables.

^14^This model was not mentioned in the pre-registration, as we assumed that model 7 would provide information regarding this interaction.
